# A Comparative Study of Precision Surface Grinding Using Additively Fabricated Acrylonitrile–Butadiene–Styrene (ABS) Wheels with Continuous and Serrated Working Surfaces

**DOI:** 10.3390/ma17235867

**Published:** 2024-11-29

**Authors:** Dawid Zieliński, Mariusz Deja, Mateusz Zator

**Affiliations:** Faculty of Mechanical Engineering and Ship Technology, Department of Manufacturing and Production Engineering, Institute of Machine and Materials Technology, Gdańsk University of Technology, G. Narutowicza Str. 11/12, 80-233 Gdańsk, Poland; mariusz.deja@pg.edu.pl (M.D.); s183843@student.pg.edu.pl (M.Z.)

**Keywords:** abrasive processes, precision grinding, grinding wheel, rapid tooling, fused filament fabrication

## Abstract

Nowadays, high requirements imposed by mechanical components make it necessary to develop modern production methods. Additive technologies have been dynamically developing in recent years, showing many advantages associated with the fabrication of elements with complex geometries and structures. One of the areas where the potential of additive technologies is exploited is the rapid tooling sector, which is based on the rapid production of tools and components used in various manufacturing methods. Currently, apart from industrial additive fabrication using metal and plastic powders, desktop and low-cost devices for additive manufacturing are gaining more and more importance in the production of functional elements. This paper presents the experimental results obtained from testing the micro-abrasive acrylonitrile–butadiene–styrene ABS tools fabricated by fused filament fabrication (FFF) technology and reinforced with SD 28/20 diamond grains uniformly distributed on the working surface of the tools after they were made. Precision surface grinding operations of 41Cr4 alloy steel were carried out on a portable five-axis CNC milling machine using wheels with continuous and serrated working surfaces. The tool with a serrated working surface enabled a more efficient material removal and produced a better surface finish. In particular, a low wear rate of both FFF-printed tools was confirmed after all experiments. Promising results were obtained, showing the potential for a wider industrial application of the tested tools.

## 1. Introduction

Additive manufacturing implies different technologies that enable quick and fast fabrication of mechanical components of complex shapes. Part fabrication is based on the application of successive layers of material, usually in powder or liquid form [1]. According to ASTM 52910 standards [2], there are seven main groups of 3D printing methods: powder bed fusion, photopolymerization, material extrusion, material and binder jetting, direct energy deposition as well as sheet lamination. These methods are differentiated by, among other factors, the type and form of material being processed, how the material is applied to the working platform, and the source of energy generated for the additive process [1,3]. The growing importance of the 3D printing sector, mainly related to the application of new materials as well as the development of efficient manufacturing processes and printing systems, makes it possible to fabricate advanced parts for applications in various industrial fields. Nowadays, methods of using metal and metal alloy powders, e.g., laser powder bed fusion (LPBF) technology, are widely used in the energy, maritime, aerospace as well as tooling industries [4,5]. LPBF-fabricated components are characterized by good and repeatable mechanical properties, which, unfortunately, also translates into high costs and time in the production process. Desktop and low-cost 3D printers using thermoplastics and based on FDM/FFF (*fused deposition modeling*/*fused filament fabrication*) methods are currently gaining more and more interest not only in amateur applications but also in industrial fields. These types of devices are also characterized by relatively simple operation [6].

The working principle of a standard FDM/FFF printer is to extrude the molten material in the form of a filament and distribute it through the printheads layer by layer on the building platform. The whole process is repeated until the full geometry of the fabricated part is achieved [7]. Currently, it is possible to process a wide range of materials with different mechanical properties, including the materials dedicated to the tool industry, which enables the design and fabrication of high-quality prototypes and functional parts. The most widely used thermoplastic materials are ABS, PLA, PA, PETG, TPU, and PEI. Standard low-cost FDM printing devices can be used to produce parts from ABS composite filaments containing synthetic microdiamond filaments [8]. 

Meeting the high demands of the modern manufacturing industry means that the finishing processes are under constant and continuous development. Recent research also highlights the growing importance of additively fabricated tools used in various machining processes. The application of ingenious variations in tool structure and the fabrication of fully and partially elastic grinding wheels were pointed out in Ref. [9] as one of the key issues in the development of abrasive processes. Due to this fact and the numerous advantages of AM-fabricated tools, such as complex internal and external geometries as well as characteristic structure and mechanical properties, different 3D printing technologies have been increasingly used for the fabrication of abrasive tools. As presented in [10], three groups of abrasive tools used in various abrasive machining processes, such as grinding, lapping, and polishing, have been currently developed. Table 1 provides an overview of the main abrasive tools based on selected 3D printing technologies, along with a short description of the building process.

Additive manufacturing methods based on laser powder bed fusion (LPBF) technology have been widely used for the fabrication of prototype grinding and polishing wheels [11]. As a result, it is possible to produce abrasive tools with precisely defined internal and external geometries [12,13], including grinding wheels with porous structures [14,15] and regular grain distribution [16,17]. One of the main limitations of such metal-bonded abrasive tools is their relatively high production costs and complex fabrication process. For soft resin tools used in grinding, lapping, and polishing, a mixture of material in the form of liquid resin and loose abrasive grains was cured with UV [18,19,20] or a laser light [21]. Recently, many studies have investigated resin-bonded grinding wheels. In order to improve the performance of the grinding process, different types of bond materials [22,23,24], as well as tool geometries and structures, have been analyzed. [25]. However, pure resin tools are characterized by low mechanical properties due to their high wear rate, which limits their use in industry. For this reason, it is necessary to use additional fillers or additives in the form of metal particles and glass or carbon fibers, which significantly complicates and extends the fabrication process of grinding wheels. Currently, more attention is paid to the fabrication of powder bed fusion abrasive tools using polyamide. For instance, a mixture of polyamide powder and diamond grains was used to produce grinding wheels with cooling holes for glass machining [26]. SLS-printed tools presented in Ref. [27] were effectively used in the machining of difficult-to-cut materials such as Al_2_O_3_ ceramics. Using abrasive paste and loose diamond grains deposited on the tool’s surface during the grinding process results in the more effective embedding of the abrasive grains into the soft and elastic surfaces of the tool. Consequently, an efficient machining process and a significant improvement in the surface finish were achieved. Furthermore, the prototype SLS-printed wheels exhibited significantly lower wear compared to resin-bonded tools and at a similar level to conventional lapping discs [28]. Material extrusion-based additive manufacturing (MEAM) may provide another approach for building prototype tools. The authors of the paper [29] proposed an FDM-based end mill tool from commercial acrylonitrile–butadiene–styrene (ABS) filament. Additively fabricated thermoplastic tools were then used to mill soft, expandable polystyrene. In another study [30], a filament consisting of zirconium-tempered alumina ceramic (ZTA) with powder particles was developed and used as a raw material for the material extrusion process. Hybrid or reinforced FDM filaments have been indicated as materials suitable for producing grinding wheels [31]. For this reason, many researchers are focusing on developing composite feedstock materials for FFF/FDM processes [32]. For example, the authors of the study [33] used a ceramic particle-reinforced ABS filament for rapid tooling. A new concept for the fabrication of ultra-fine grinding tools based on hybrid filament consisting of polyamide PA12, ZrO_2_ particles, and diamond grains was demonstrated by researchers in [34]. The experimental tests indicated a significant reduction in the surface roughness of fused silica samples after machining.

As the abrasive tools produced by FFF technology and based on virgin ABS material have not been studied so far, an attempt was made to design and fabricate micro-abrasive tools which can be used along with PCD-based slurry for precision surface grinding. This paper presents some important effects of precision grinding performed on annealed 41Cr4 alloy steel workpieces of 45HRC hardness using micro-abrasive tools with continuous and serrated active surfaces. The obtained results showed that the abrasive tool with the serrated working surface allowed for more efficient material removal and produced a better surface. In particular, the examination of the worn tool confirmed the lower wear rate of both FFF-printed tools.

## 2. Materials and Methods

### 2.1. Tool Design and Fabrication

**Figure 1:**
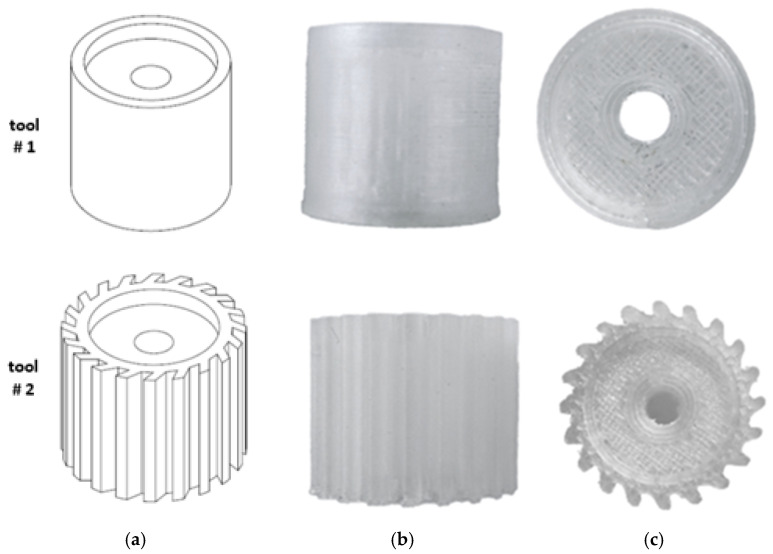
Prototype tools made by FFF: (**a**) CAD models, (**b**) front, and (**c**) top view of fabricated parts.

**Figure 2:**
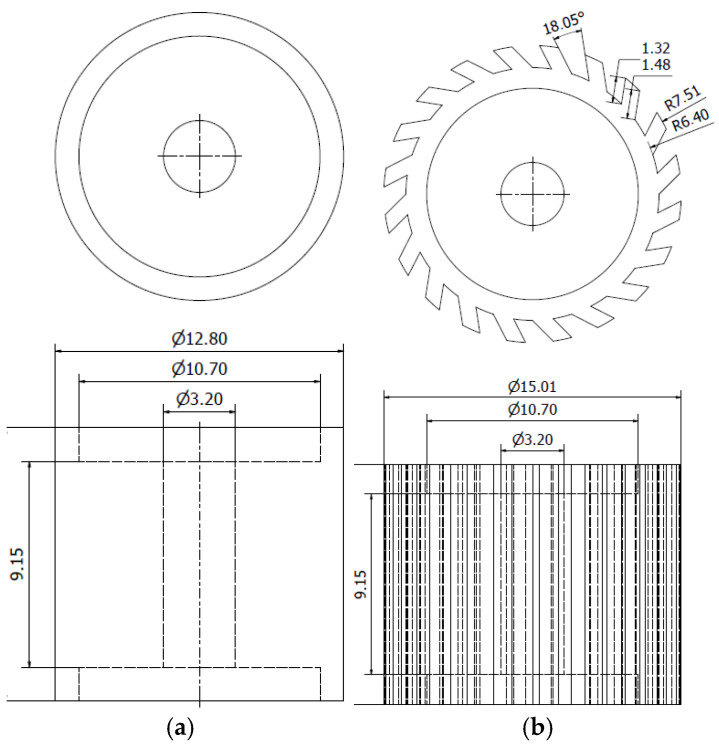
Specific parameters of the wheels made by FFF: (**a**) tool #1, (**b**) tool #2.

**Figure 3:**
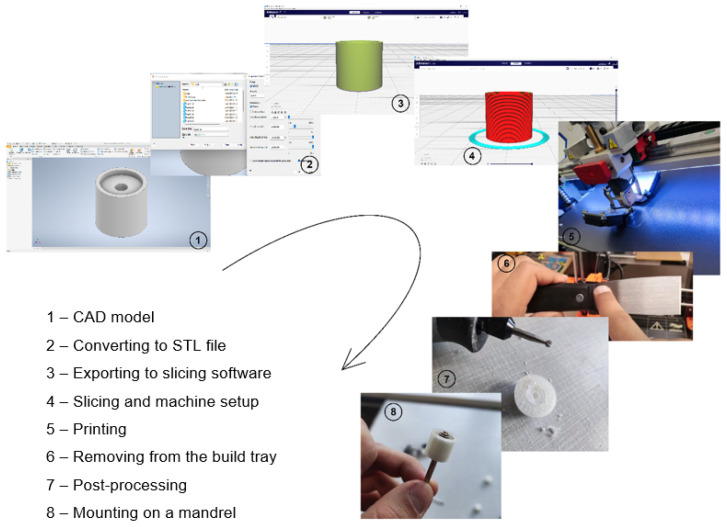
Rapid prototyping process chain of FFF-printed tools.

In this study, two abrasive tools with smooth and serrated external surfaces fabricated by FFF technology from ABS filament (3DGence Sp. z o. o., Przyszowice, Poland, ϕ1.75) were tested in grinding operations. Tool #1 had a smooth cylindrical shape with both assumed diameter and length equal to 13 mm, while tool #2 had a number of linear grooves along its cylindrical surface (Figure 1). The protrusions and the voids (empty spaces) between them were to serve as reservoirs for the abrasive paste added before machining. The specific parameters of the grinding wheels used in this study are presented in Figure 2. The FFF fabrication was carried out on a 3D printer, 3DGence ONE (3DGence Sp. z o. o., Przyszowice, Poland), keeping the process parameters specified in Table 2, following the steps shown in Figure 3.

### 2.2. Experimental Setup

**Figure 4:**
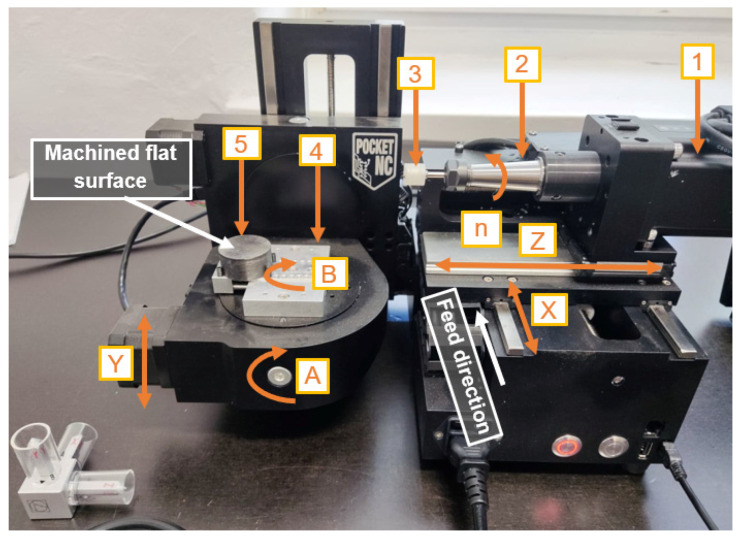
Pocket NC portable five-axis CNC milling machine tool used in grinding tests: 1, steeper motor; 2, spindle; 3, FFF-printed tool; 4, vise; 5, workpiece; X, Y, and Z, linear axes; A and B, rotary axes; *n*, spindle rotations.

**Figure 5:**
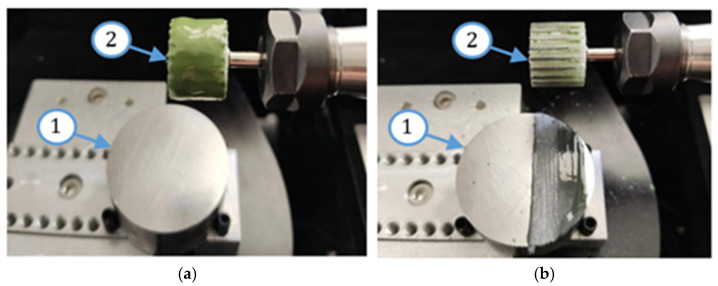
Workpiece (1) and FFF-printed tool #2 (2) with abrasive paste deposited on its cylindrical surface before (**a**) and after (**b**) the first pass using FFF-printed tools.

In this experimental study, flat surfaces of 41Cr4 alloy steel workpieces (Gdańsk University of Technology, Gdańsk, Poland) were grounded after pre-machining on a double-disk grinder (IWF TU Berlin, Institute for Machine Tools and Factory Management, Technische Universität Berlin, Berlin, Germany) to provide good plane parallelism between both flat surfaces as shown in Refs. [35,36]. Grinding tests were performed on a Pocket NC portable five-axis CNC milling machine (Penta Machine Company, Belgrade, MT, United States) presented in Figure 4. The printed tool (3) was mounted in a spindle (2) driven by a stepper motor (1). The sample (5) was clamped in a vice (4) through its cylindrical surface. The radial depth of the cut was set along the *Y* axis, and the feed rate was in the direction of the *X* axis. The grinding tests were performed keeping the process parameters presented in Table 3. The three theoretical radial depths of cut, i.e., *a_e–t_* = 0.1; 0.2; 0.3 mm, were programmed in the CNC controller. Before each of the three tests, a thin layer of paste with SD 28/20 diamond grains and 20% volumetric concentration (Pasta-Diamentowa.pl, Ełk, Poland, according to the standards PN75/M59108 [37]) was deposited in the amount of 1 mL on the tools (see Figure 5).

**Figure 6:**
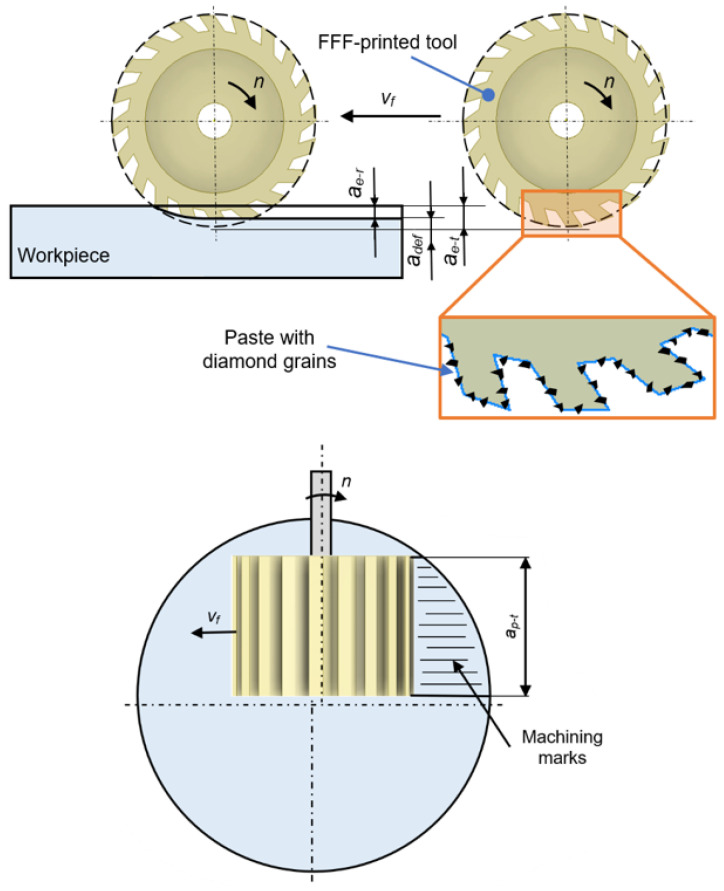
Illustrative kinematic diagram of the performed process using FFF-printed tools with radial *a_e–t_* and axial *a_p–t_* depths of cut.

The measurements carried out after experiments revealed that the real radial depth of cut *a_e–r_* during machining was smaller than the theoretical depth *a_e–t_* due to the elastic deformation *a_def_* of the tool in the contact zone, which is schematically shown in Figure 6. The tool deformation causes the diamond grains to be pressed and permanently fixed on the tool’s working surface layer. The grinding process was performed using the kinematics corresponding to up-milling. In the first test, six consecutive passes of the tool were carried out at the same depth of cut adjusted by means of the CNC program to *a_e–t_*_1_ = 0.1 mm. The plane parallelism between both flat surfaces achieved by double-sided grinding ensured equal material allowance for each pass of the tool. After each working pass, the tool was raised above the machined surface and moved to the starting point at a rapid feed rate. In the second test, this procedure was repeated six times for the next two depths, i.e., for *a_e–t_*_1_ = 0.1 mm and *a_e–t_*_2_ = 0.2 mm, and in the third test for all three depths selected, i.e., for *a_e–t_*_1_ = 0.1 mm, *a_e–t_*_2_ = 0.2 mm, and *a_e–t_*_3_ = 0.3 mm. The theoretical axial depth of cut *a_p–t_* = 13 mm was the same as the tool length (Figure 6). The mass loss of the steel samples was determined using an Axis ACA520 laboratory balance (AXIS Sp. z o. o., Gdańsk, Poland) with a resolution of 0.001 g, while the surface roughness was measured using a contact profiler HOMMEL TESTER T500 (Hommelwerke GmbH, Villingen-Schwenningen, Germany) according to the DIN4777 standards [38]. The surface topography of the worn tool was measured using a 3D optical profiler, S neox Sensofar (Sensofar, Barcelona, Spain), according to the ISO25178 standard [39]. The analysis of the obtained surface topographies was carried out using SensoMAP Premium 9.1 software. The indicated experimental tests were not repeated, but measurements of surface roughness were repeated three times after each test. 

## 3. Results of Machining Experiments

**Figure 7:**
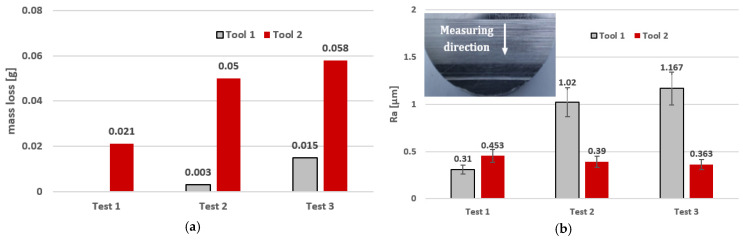
Mass loss of the steel samples (**a**) and average values of the Ra surface roughness parameter (**b**) after machining using FFF-printed tools.

As can be seen in Figure 7, tool #2 with a serrated working surface guarantees a more efficient material removal. The reason is that the grooves around the external surface of tool #2 act as reservoirs for the accumulation of diamond abrasive paste. Due to this fact and the tool deformation in the machining zone, a better reinforcement with abrasive grains was possible. The smooth cylindrical surface of tool #1, devoid of cavities and voids, causes an easier removal of the abrasive paste during machining, and thus, after the first test, no material loss was observed. Additional tool passes performed in the second grinding test, with two radial depths of cut, i.e., *a_e–t_*_1_ = 0.1 mm and *a_e–t_*_2_ = 0.2 mm, doubled the material removal when tool #2 was used, and already made it possible to perform machining with tool #1, but with a very low material loss. Consequently, there was a low increase in the material removal for both tools in the third grinding test, performed at three radial depths of cut, i.e., *a_e–t_*_1_ = 0.1 mm, *a_e–t_*_2_ = 0.2 mm, and *a_e–t_*_3_ = 0.3 mm. The use of tool #2 resulted in both greater material loss and lower surface roughness, which can be seen in Figure 7a and Figure 7b, respectively. The value of the Ra roughness parameter decreased from Ra = 0.453 µm after the first test to Ra = 0.36 µm after the last test. This was due to the fact that the grains became embedded deeper on the serrated working surface, resulting in the largest theoretical radial depth of cut for test #3. The opposite situation took place for tool #1, for which an increase in the surface roughness to the value of Ra = 1.167 µm was observed after subsequent tests with low material removal. Most of the grains were removed from the smooth cylindrical surface, but the remaining grains increased the roughness under the biggest theoretical radial depth. The lack of grooves on the tool’s working surface caused them to exert more pressure on the workpiece.

**Figure 8:**
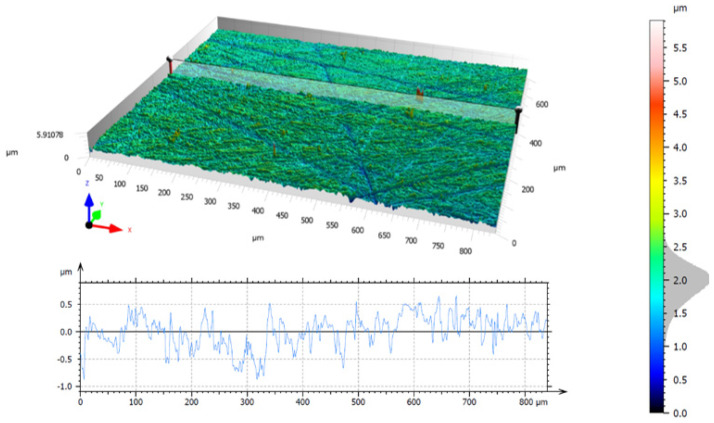
Surface topography of the sample after double-face grinding and before machining tests using prototype FFF-printed tools.

**Figure 9:**
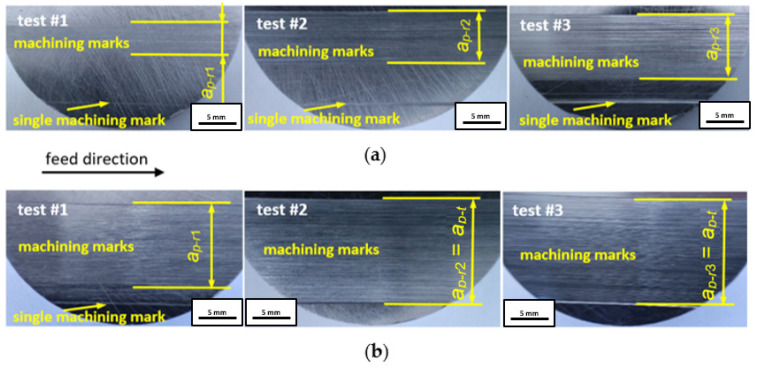
Machining marks on steel samples after tests using tools with a cylindrical circumferential surface (**a**) and with protrusions around the circumference (**b**).

**Figure 10:**
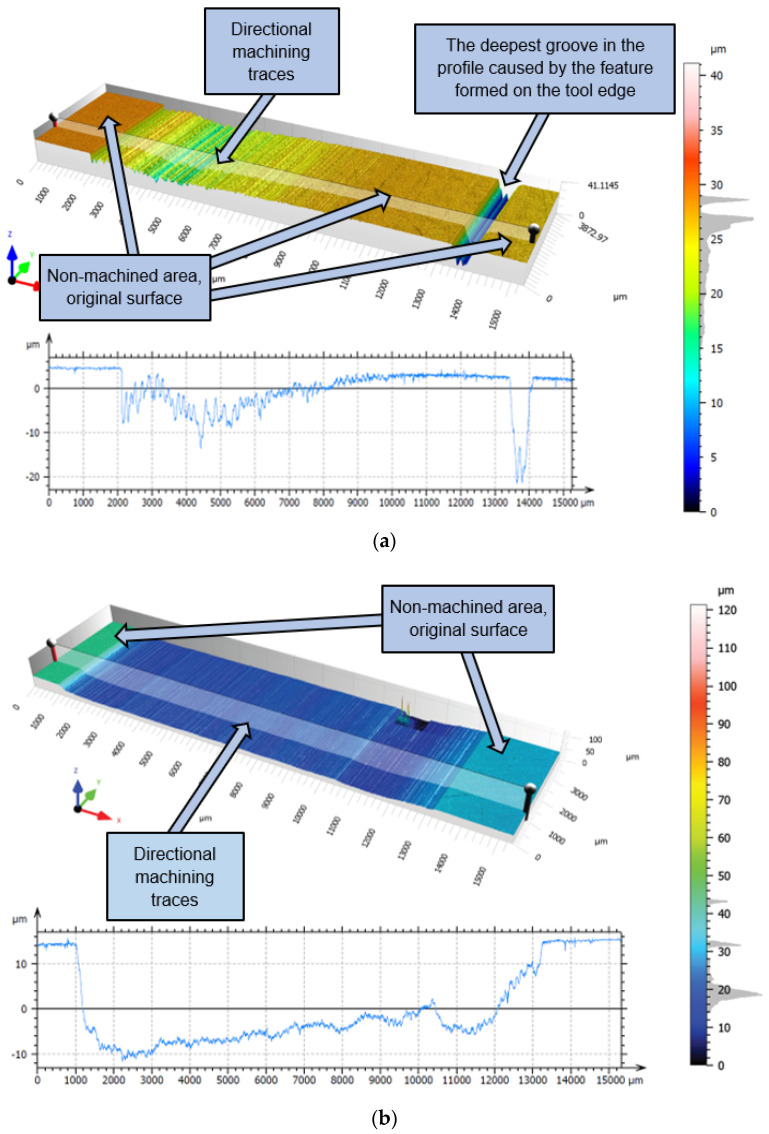
Surface topographies and profiles recorded on steel samples after performing test #3 using FFF-printed tool #1 with a cylindrical circumferential surface (**a**) and #2 with grooves around the circumference (**b**).

**Figure 11:**
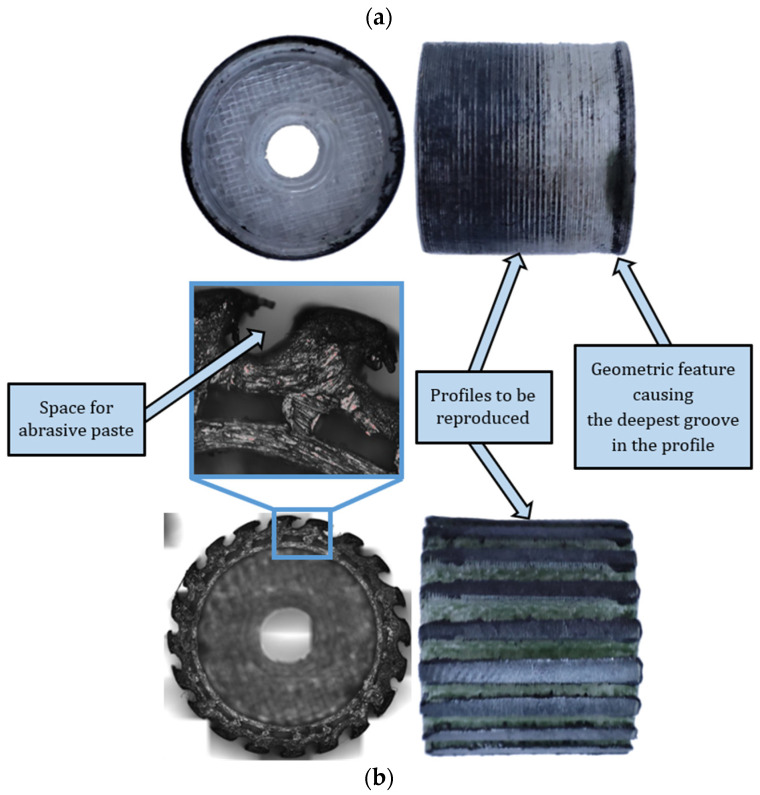
Specific features of worn tools after performing all tests: (**a**) tool #1 with a cylindrical circumferential surface (**top**), (**b**) tool #2 with grooves around the circumference (**bottom**).

The topography of the ground surface before tests, with microgrooves after double-face grinding, is shown in Figure 8. The topography with non-directional microgrooves resulted from planetary kinematics of the grinding process and allowed for easy distinguishing between original and newly machined areas, with newly created microgrooves and marks. After each test, the machining marks produced on the ground surfaces were observed in the direction of the feed movement, as depicted in Figure 9 and Figure 10. Measured topographies containing the machining marks left by the abrasive grains, along with extracted exemplary profiles after the third test using tools #1 and #2, are presented in Figure 10a and Figure 10b, respectively. It was assumed that the theoretical axial depth of cut *a_p–t_* = 13 mm was the same as the tool length (Figure 6). As is seen in Figure 9b, this requirement was met only for tool #2 and tests #2 and #3. The extracted profiles can be used to determine both the real radial and axial depths after individual passes. The depth of the microgrooves formed confirms that the material removal efficiency was distinctly better for tool #2. The depth of single machining marks varied significantly, which probably resulted from uneven contact of elastic tools with the machined surface. As shown in Figure 10a, a non-machined area appears between machining marks. The deepest groove in the tool profile was created by the geometrical feature formed on the tool edge, as shown in Figure 11a. The use of tool #2 resulted in a significantly wider, deeper, and more even groove profile, with the assumed axial depth of cut, compared to a profile obtained by tool #1. More advanced characterization of the surface topographies produced by cutting and abrasive operations using areal (3D) roughness parameters can be found in Ref. [40].

## 4. Discussion

After each test, machining marks were observed in the direction of the feed movement. For tool #1, the achieved axial depth of cut *a_p–r_* increased after each test but never reached the assumed value of *a_p–t_* = 13 mm, as shown in Figure 10a. This effect is caused by uneven material removal resulting from the shape deviation of the tool’s working surface, resulting, in turn, in the uneven distribution of the paste on the tool and the machined surface during machining (Figure 5b). However, as shown in Figure 10b, it was possible to achieve the assumed axial depth of cut in the grinding tests when using tool #2. This was caused by an easier deformation of tool #2, equipped with grooves around its cylindrical external surface, thus allowing for a more accurate adjustment of the tool shape to the configuration of the machined surface. 

**Figure 12:**
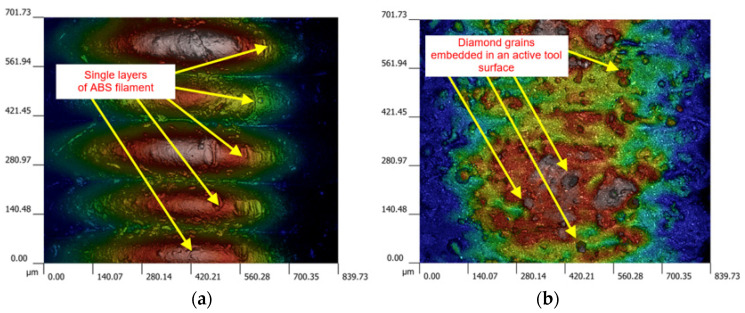
Working peripheral surface of an FFF-printed tool #1 before machining (**a**) and after completing the third test with diamond grains embedded in the surface (**b**).

As a result of reproducing the actual shape of the tool shown in Figure 11, the characteristic profiles containing a series of single microgrooves were created by diamond grains embedded in the top layer of the tool material demonstrated in Figure 12. Moreover, for the complex geometry of tool #2 presented in Figure 5b, the spaces between protrusions were able to accumulate the diamond abrasive paste to perform effective material removal even up to 36 working passes. This resulted in a longer tool life of the grooved grinding wheel. The obtained results, as well as the macro- and microscopic observations, did not show excessive wear of the tool, but a detailed wear analysis with some quantitative data will be conducted in further systematic research. 

The application of different AM methods is a novel approach to the fabrication of grinding wheels with strictly defined external and internal geometries. Currently, tools based on metal powders and their alloys are successfully used in the production of grinding wheels with high porosity and regular grain distribution. On the other hand, fabrication and post-processing are much more difficult, longer, and more expensive than resin- or filament-based methods. Resin-bonded wheels made of pure resins are characterized by relatively low mechanical properties, which significantly limits their range of applications, especially for difficult-to-cut materials. However, inserting additional fillers extends the process and cost of making the tool. The wheels proposed by the authors with a strictly defined structure are cheap and quick to produce using an easy-to-use, low-cost 3D printer and commercially available ABS filament. In contrast to the tools tested by many other researchers, an abrasive paste containing diamond grains was applied to the working surface of the tool only after the wheels were made. Moreover, the designed protrusions on the working surface enabled the appropriate retention of grains on the surface of the tool, which was confirmed by the experimental results presented in this paper. In general, AM-fabricated wheels can be an alternative to conventional abrasive tools and can only be used in unit or small-batch production. Usually, the overall production costs of AM tools are lower, while the process requires the use of fewer operations. In addition, 3D printing enables the fabrication of tools with complex internal and external geometries that are difficult to achieve using conventional subtractive methods. These types of structures or features can affect the increase in grinding performance. Nevertheless, AM-printed wheels, especially from plastic material, display significantly lower mechanical properties than conventional grinding wheels, which limits their wider industrial application. Other challenges include obtaining reproducible features in the form of designed structures, as well as reducing the large number of defects occurring during the printing process. Thus, the amount of experimental data with prototype AM tools is still limited and requires further systematic studies, taking into account specific industrial conditions.

## 5. Conclusions

Results of this study devoted to grinding steel surfaces using FFF-printed tools allow for the formulation of the following conclusions:

FFF method allowed for rapid and chip fabrication of prototype tools from commercial and virgin feedstock material acrylonitrile–butadiene–styrene (ABS) of different designs used after their reinforcement with SD 28/20 diamond grains for machining flat surfaces of 41Cr4 alloy steel workpieces after annealing process (hardness 45HRC). Microscopic observations confirmed the presence of diamond grains embedded into the tool’s working surface.

The novel design of the wheel includes strictly defined features in the form of protrusions and voids on the working surface, serving as the reservoirs for the abrasive paste. The geometry of the tool’s working surface significantly influences the obtained technological effect. The tool with the serrated active surface (tool #2) allows for more efficient material removal and produces a better surface finish. This is because the spaces between the grooves around the circumference serve as reservoirs for the abrasive paste.

Reproducing the actual shape of the tools on the machined surfaces causes the profiles with single microgrooves to be created by diamond grains embedded in the tool body. The depth of single machining marks varies significantly, which results from uneven contact of elastic tools with the sample’s machined surface.

The tools applied in this study are not able to provide uniform surface shapes with assumed radial depths of cut, mainly due to the elastic deformation of the tool. The shape deviation of the tool and geometric features on the working surface of the tool, newly created during machining, result in uneven material removal. However, the serrated tool allows for achieving the assumed axial depth of cut due to the easier adjustment of its shape to the machined surface and better reinforcement with abrasive grains.

The tools used were characterized by a relatively low wear rate and a long lifespan. Microscopic observations reveal that the gaps between protrusions were not erased after all tests were performed, which allowed for the abrasive medium to be accumulated for performing even up to 36 working passes.

Since the machined surface is reproduced by the shape of the working surface of the tool, the machining of free-form surfaces seems to be a promising and potential area for further research. The influence of dimensional and shape accuracy of the fabricated tools on the accuracy of machined features will also be thoroughly studied.

Further improvement in the performance of the additively fabricated abrasive wheels using different AM methods should focus on a wider study of the effects of printing parameters on the tools’ quality and their mechanical properties. Another possibility is the development of new hybrid filaments and the determination of the appropriate conditions for their production and processing using an extrusion-based method. The design of some features on the working surface, serving as the reservoirs for the abrasive, could improve the working performance that was actually shown in this research.

## Figures and Tables

**Table 1:** Main groups of abrasive tools fabricated additively based on reference [10].

Type of Abrasive Tools and Fabrication Method	Characteristics of the Building Process	Possible Application Area
Metal-bonded tools based on LPBF processes	Mixture of metals and their alloys in powder form and abrasive grains melted by laser beam	Grinding and polishing
Resin-bonded tools based on UV-curing resin processes	Mixture of liquid resins and abrasive grains cured with UV light	Grinding, lapping, and polishing
SLS (selective laser sintering)-printed tools	Mixture of polyamide in powder form sintered by laser beam with grains added during or after the sintering process	Grinding and lapping

**Table 2:** Set of FFF process parameters used for the fabrication of tested tools.

Printer	3DGence ONE
Raw material	ABS in filament form
Support material	same as model
Module (nozzle diameter)	0.4 mm
Filament diameter	1.75 mm
Layer height	0.15 mm
Infill per cent	40%
Bed adhesion	Brim
Model material temperature	250 °C
Post-processing	Removal from the build tray after printing process; removal of support material using a manual grinder

**Table 3:** A set of grinding parameters used in the machining tests.

Test No.	Spindle Speed *n* [rpm]	Feed Rate *v_f_* [mm/min]	Theoretical Radial Depth of Cut *a_e–t_* [mm]	Number of Tool Passes
1	4000	200	*a_e–t_*_1_ = 0.1	6
2	4000	200	*a_e–t_*_1_ = 0.1	6
	4000	200	*a_e–t_*_1_ = 0.2	6
3	4000	200	*a_e–t_*_1_ = 0.1	6
	4000	200	*a_e–t_*_1_ = 0.2	6
	4000	200	*a_e–t_*_1_ = 0.3	6

## Data Availability

Dataset available on request from the authors.
